# The Ethics of Gum-Chewing

**Published:** 1892-06

**Authors:** Kate I. Dein


### The Ethics of Gum-Chewing.

Almost constantly you meet women or girls gently moving their jaws to and fro as calmly as cows chewing their cuds. Every time you board a street car you will find two or three young women dreamily gazing out of the windows and mechanically working their jaws.

There is an individuality about the manner of chewing gum. No two women chew exactly alike. Some women chew noisily and others quietly; one will chew with her mouth closed, another with it open. A thin, nervous woman chews quickly and almost nervously. She chews gum just as she does everything else. A calm, self-possessed woman chews slowly and almost imperceptibly. You might sit beside her for a quarter of an hour before finding out that she was chewing at all. An indecisive woman slowly one moment and quickly the next with a lateral movement of the jaw. The cautious woman moves her jaws slowly and regularly by straight up-and-down using. The vivacious woman chews and talks and talks and chews, and the worst of it is you can not keep from watching her. You are tempted to count how often her jaw rises and falls per second. You try to look the other way and can't. You keep your eyes on her until your brain fairly reels. It is maddening to watch a talkative woman when she is chewing gum. Then, no woman has ever been able to look pretty and chew gum at the same time. Nor is it for want of practice, for a gum-chewer chews when she dances, walks, rides or talks. She never stops except to eat and sleep.

While there are many objections to gum-chewing, expense is not one. An economical girl can stretch out a piece of gum so far that it will last all winter. The older the gum is the more she becomes attached to it. She knows its strong points by that time. Some women like a variety, though. I have found as high as ten pieces of gum under the edge of one table.

Under the edge of the table is a favorite place for the safe-keeping of gum, though window-ledges, shelves, chair-backs, etc., are considered fair places.

There is, however, one redeeming feature; when you see a woman chewing gum in a street car, on the street, in a store or any public place, if you can't tell who she is, you can tell what she isn't. She is not a lady. You can never make a mistake about that. There are some things that a lady prizes more than money and self-indulgence. She places a value on herself. The girl who chews cheapens herself in the eyes of all who see her. —*Kate I. Dein.*

